# TSP-GNN: a novel neuropsychiatric disorder classification framework based on task-specific prior knowledge and graph neural network

**DOI:** 10.3389/fnins.2023.1288882

**Published:** 2023-12-21

**Authors:** Jinwei Lang, Li-Zhuang Yang, Hai Li

**Affiliations:** ^1^Anhui Province Key Laboratory of Medical Physics and Technology, Institute of Health and Medical Technology, Hefei Institutes of Physical Science, Chinese Academy of Sciences, Hefei, China; ^2^University of Science and Technology of China, Hefei, China; ^3^Hefei Cancer Hospital, Chinese Academy of Sciences, Hefei, China

**Keywords:** neuropsychiatric disorders, task-specific prior knowledge, brain decoding, functional connectivity, graph neural network

## Abstract

Neuropsychiatric disorder (ND) is often accompanied by abnormal functional connectivity (FC) patterns in specific task contexts. The distinctive task-specific FC patterns can provide valuable features for ND classification models using deep learning. However, most previous studies rely solely on the whole-brain FC matrix without considering the prior knowledge of task-specific FC patterns. Insight by the decoding studies on brain-behavior relationship, we develop TSP-GNN, which extracts task-specific prior (TSP) connectome patterns and employs graph neural network (GNN) for disease classification. TSP-GNN was validated using publicly available datasets. Our results demonstrate that different ND types show distinct task-specific connectivity patterns. Compared with the whole-brain node characteristics, utilizing task-specific nodes enhances the accuracy of ND classification. TSP-GNN comprises the first attempt to incorporate prior task-specific connectome patterns and the power of deep learning. This study elucidates the association between brain dysfunction and specific cognitive processes, offering valuable insights into the cognitive mechanism of neuropsychiatric disease.

## 1 Introduction

Neuropsychiatric disorder (ND) defines a wide range of psychiatric symptoms accompanying specific emotional, memory, social, or other cognitive impairments (Eddy, 2019; Porcelli et al., 2019; Jahn et al., 2021). Different subtypes of diseases, such as attention-deficit/hyperactivity disorder (ADHD) (Zepf et al., 2019), autism spectrum disorder (ASD) (Vaidya et al., 2020; Wadhera and Kakkar, 2020), and schizophrenia (SZ) (Ioakeimidis et al., 2022; Riedel et al., 2022) show abnormal brain activity during specific task context compared to healthy controls. Mental disorder diagnosis using neuroimaging and machine learning is thus promising (Lanillos et al., 2020; Perez et al., 2021).

Recent years have seen explosive growth in applying deep learning to facilitate ND classification (Chan et al., 2019; Canario et al., 2021; Liu et al., 2021). Previous studies often use brain functional connectivity (FC) or graph theory features (Farahani et al., 2019) and build convolutional neural networks (CNNs) for disease classification (Kim et al., 2016; Guo et al., 2017). However, brain networks are generally irregular and non-Euclidean structures, which can be better captured by graph neural networks (GNNs) than CNNs (Parisot et al., 2017; Zhang et al., 2020; Li L. et al., 2021; Li X. et al., 2021; Zhao et al., 2022). The benefit of GNN is due to the peculiarities of the message-passing mechanism on the graph (Ying et al., 2019). A pioneering study by Parisot and colleagues integrated the FC matrix and phenotype information to construct a sparse graph that captures participants’ relationships (Parisot et al., 2017). Subsequently, various graph structures (Li L. et al., 2021) and graph modules, such as graph pooling (Li X. et al., 2021) and even dynamic graph strategies (Zhao et al., 2022), have been proposed, significantly enhancing GNN models for neuropsychiatric disease classification. These models utilize node pooling or edge convolution layers to selectively aggregate important node features, thereby providing insights into relevant diseases from a regional perspective within the brain. For example, default mode network (DMN) and memory-associated brain regions have been identified as biological markers of ASD (Li X. et al., 2021), while damage to the DMN associated with occipital and frontal lobes may explain ADHD (Zhao et al., 2022).

The whole-brain resting-state FC matrix contains redundant and spurious correlations because of confounding or collider effects (Sanchez-Romero and Cole, 2021). It is thus valuable to extract and define distinct connectivity patterns specific to certain cognitive contexts. Recent studies have demonstrated that task-state FC patterns play an essential role in dynamically reshaping brain networks and modulating the flow of neural activity during task performance (Cole et al., 2021; Hearne et al., 2021). These task-related changes in brain network activity provide valuable prior knowledge for understanding the mechanisms underlying brain disorders (Briend et al., 2019; Xia et al., 2019; Kofler et al., 2020; Riedel et al., 2022). However, previous research on ND classification often overlooked this valuable prior information (Gupta et al., 2022; Jiang et al., 2022).

Decoding studies on brain-behavior relationships provide an insightful framework (Jiang et al., 2020; Finn, 2021). We hypothesize that incorporating prior knowledge of task-specific connectivity patterns can improve the performance of ND classification. Motivated by the underlying association between brain decoding and disease diagnosis, the present study seeks to integrate task-specific prior (TSP) knowledge (task-specific functional connectivity) and GNN into a ground-breaking framework for detecting neuropsychiatric disease, dubbed TSP-GNN. We use the Elastic-Net regression model to decode task-specific brain connectome patterns from task-state fMRI in healthy people. Then, task-specific connectome patterns were migrated to illness classification using resting-state fMRI. Finally, we build a population-based graph convolution network to detect brain disease in two neuropsychiatric datasets. The brain decoding approach reduces the dimension of the brain network while providing interpretive information relevant to the task context. Our results demonstrate that task-specific connectome improves disease categorization compared to whole-brain nodes and sheds light on the relationship between brain pathology and specific cognitive processes.

## 2 Materials and methods

### 2.1 Participants

#### 2.1.1 HCP dataset

The Human Connectome Project (HCP) (Van Essen et al., 2013) is a remarkable and widely available dataset aimed at defining the anatomical and functional interconnection of the human brain. This dataset contains high-resolution structural MRI, resting-state fMRI, task fMRI scans, and detailed behavioral information for over 1,000 healthy individuals. Subjects completed seven scanner tasks: motor execution, language, emotion, social cognition, working memory (WM), relational, and gambling-related processes. The seven tasks, which lasted for about 20–30 frames under different conditions during each block, and the detailed task paradigm were described in Supplementary Table S1.

#### 2.1.2 Neuropsychiatric dataset

The present study consisted of two datasets, ADHD^1^ and ABIDE,^2^ for the investigation of disease classification. The ADHD dataset consists of eight cohorts of structural MRI and resting-state fMRI scans (Bellec et al., 2017). Similarly, the ABIDE dataset has the same acquisition modalities from 20 data sites (Cameron et al., 2013). To address the potential impact of heterogeneity in equipment and scanning parameters across different sites, we selected five data sites for the ADHD dataset and three for the ABIDE dataset. Demographic information for the two datasets mentioned above can be found in Table 1.

**Table 1:** Demographic and clinical characteristics of ADHD and ABIDE datasets.

Clinical Phenotype	HCP	ADHD			ABIDE		
	*n* = 473	TD (*n* = 239)	ADHD (*n* = 220)	*P* Value	TD (*n* = 201)	ASD (*n* = 155)	*P* value
Age (years)	28.8 ± 3.69	11.2 ± 2.58	10.9 ± 2.48	0.315	15.05 ± 5.24	14.21 ± 4.32	0.110
Gender (M/F)	227/246	122/117	164/56	< 0.001	164/37	134/21	0.218
FIQ	–	–	–	–	110.67 ± 12.77	107.29 ± 15.94	0.032
PIQ	–	–	–	–	107.58 ± 12.62	103.89 ± 15.63	0.017
VIQ	–	–	–	–	109.44 ± 12.91	106.98 ± 16.32	0.126

Age value computed using two-sample Student’s t-test with two tails; Gender value computed using chi-square test; FIQ, Full-scale IQ; PIQ, Performance IQ; VIQ, Verbal IQ.

### 2.2 fMRI data preprocessing

To ensure the reproducibility of our investigation, we utilized preprocessed fMRI results from ConnectomeDB as a basis for our subsequent analysis. We applied restricted data usage to exclude any influence of inter-individual synchronization among participants within the same family, and finally, 473 unrelated individuals were included. Additionally, we obtained two neuropsychiatric datasets that offered a standard preprocessing workflow. These datasets were directly accessible from their respective data buckets. The preprocessing of fMRI data involves numerous steps to clean and standardize the data prior to statistical analysis. All preprocessing is conducted using fMRIPrep (Esteban et al., 2019), a best-in-breed workflow that ensures high-quality preprocessing to address the challenges of robust and reproducible fMRI data preparation. The minimal preprocessing steps defined by fMRIPrep include motion correction, field unwarping, normalization, bias field correction, and brain extraction.

Subsequently, we conducted a fist-level analysis on each task-state fMRI within HCP using the general linear model (GLM). Our study used the ‘3dDeconvolve’ command in AFNI v20.3.02 to perform first-level GLM analysis. Specifically, the ‘-stim_times_FSL’ parameter was used to specify the timing of stimulus events, while the ‘-stim_file’ parameter was employed to include six head motion parameters. The ‘-mask’ parameter was also used to specify the brain mask generated by fMRIprep. The total number of stimuli ‘-num_stimts’ represented the sum of task conditions and head motion directions. All these parameters collectively constitute the design matrix for each task type, which consists of columns for each condition, nuisance variables, and a constant term, with rows corresponding to each time point of the fMRI data acquisition. The specifics of the design matrix vary according to the exact nature and timing of the task conditions within each of the seven tasks in the HCP dataset. After GLM analysis, we obtained the distribution of brain activation under different task conditions and the purified fMRI time series, devoid of noise signals from task events and motion parameters, which can enable us to investigate the neural correlates of the tasks accurately (Spencer et al., 2022).

We utilized the ‘3dNetCorr’ command by AFNI v20.3.02 to calculate the FC matrixes for both HCP and neuropsychiatric datasets based on the fMRI time series residual preprocessed by GLM. The command will calculate the correlation matrix between the time series of each pair of ROIs defined by parameter ‘-in_rois.’ The average time series and the functional connections between brain regions can be found in the destination file. The atlas adopted in our research was the Brainnetome Atlas (Fan et al., 2016), which has been extensively employed in various clinical studies (Li et al., 2020; Lee et al., 2021). The atlas consists of 246 distinct brain areas that have been carefully delineated. These brain regions can be parcellated into eight functional subsystems (Jiang et al., 2020; Lee et al., 2021). For more details on the names of brain regions in the atlas and their corresponding network allocation, please refer to Supplementary Table S2.

### 2.3 HCP behavioral performance

Due to the HCP dataset consisting of seven task fMRI scans covering various cognitive abilities, we employed corresponding performance measures as markers of these abilities. For the social task, we used the ratio of precious divided by the median response time (median_RT) under random mode. Working memory ability was evaluated using the accuracy (Acc) divided by the Median_RT score under the 2-back conditions. Emotion reflection performance was assessed using the Acc/Median_RT ratio. In the language task, the story condition was selected to indicate language competence, as performance under both story and math conditions showed a substantial association. However, no significant performance-related markers were detected for the gambling and motor tasks. We used the delay discounting measure to approximate the gambling task performance involving impulsive decision-making. Specifically, we calculated the difference in the area under the curve (AUC) scores between DDisc_AUC_40k and DDisc_AUC_200 as the gambling task score (Cai et al., 2020). A smaller AUC value indicates a higher degree of decision impulsivity. For the motor task, which does not quantitatively reflect participants’ athletic ability, we substituted the endurance measure obtained from the NIH Toolbox 2-Minute Walk Test.

In addition to the task-based fMRI, we considered resting-state fMRI, which reflects a baseline state of cognitive ability without task requirements. We utilized general ability (intelligence) measures related to reasoning, problem-solving, abstract thinking, planning, and learning. These measures, which reflect individual cognitive skills like brain fingerprint, were combined into a general factor score using exploratory factor analysis (Dubois et al., 2018; Thiele et al., 2022). Task performance indicators and their corresponding calculations for all fMRI tasks mentioned above can be found in Supplementary Table S3.

### 2.4 Task-specific functional connectome decoding based on corresponding behavioral performance

Acknowledging the advantages of the task-state connectome in predicting cognitive traits, we constructed eight models to decode task-specific brain connectome patterns across various fMRI tasks. By incorporating task performance as a driving factor, we aimed to reveal the brain connectivity patterns that contribute to cognitive traits and potentially improve our understanding of the neural mechanisms underlying these traits. Considering the superior performance of classical linear regression methods in terms of computational efficiency and their ability to capture complex brain-behavior relationships (Sui et al., 2020; Kim et al., 2021), we developed a task performance-driven brain decoding model utilizing the Elastic-net algorithm:

(1) $$\underset{\beta }{\min}\sum_{i=1}^{n}{\left(f\left(x_{i}\right)-y_{i}\right)}^{2}+\lambda \sum_{j=1}^{p}\left(\alpha \left|\beta_{j}\right|+\frac{1}{2}\left(1-\alpha \right){\left\|\beta_{j}\right\|}^{2}\right)$$

**Figure 1:**
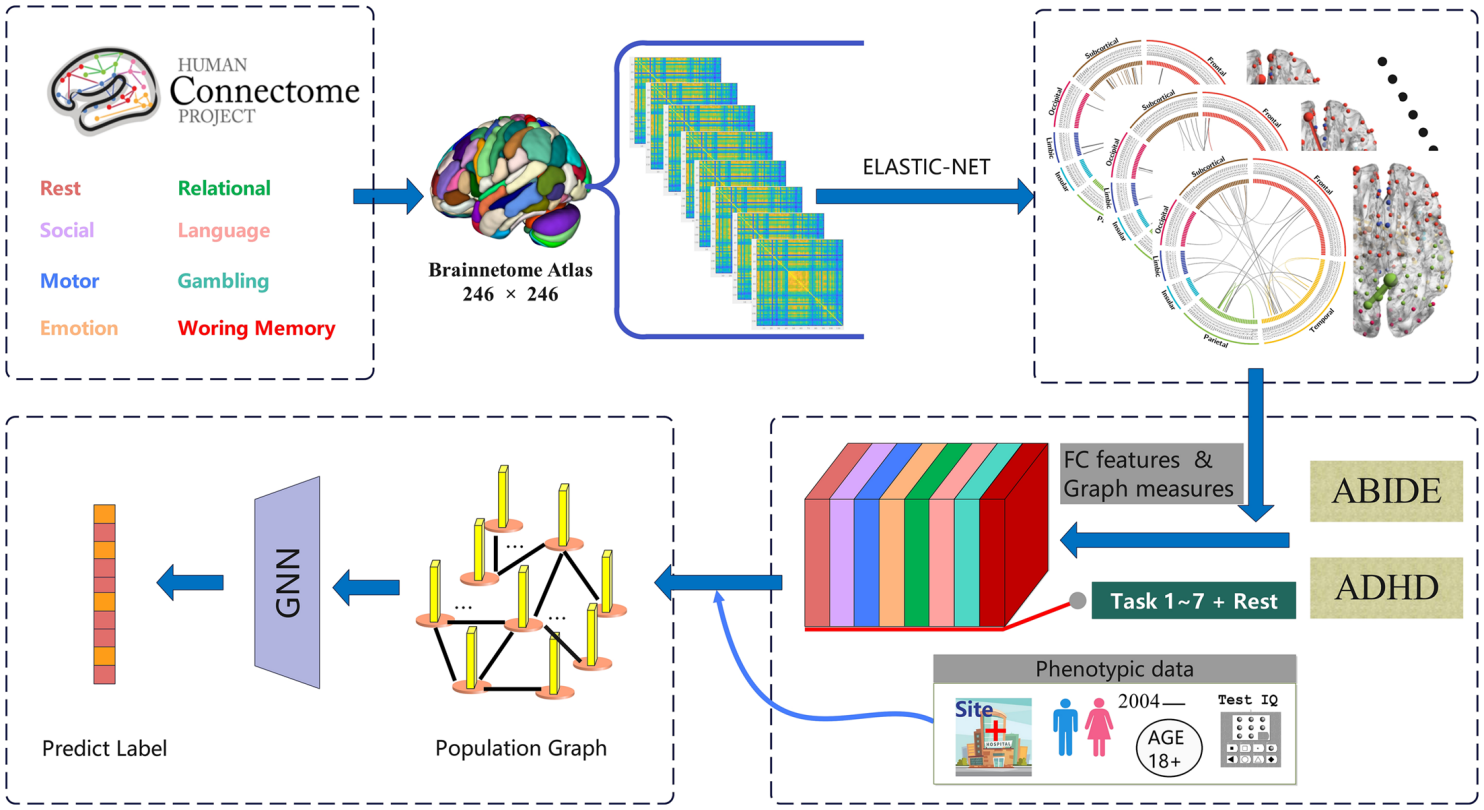
The architecture overview of the proposed TSP-GNN framework which combines task-specific patterns for disease diagnosis. The top half of the framework decodes task patterns based on cognitive performance, while the bottom extracts task-specific functional connectome and graph theoretical measures from various disease datasets. Subsequently, phenotypic information is integrated to construct the population-based graph neural network to achieve disease classification.

The Elastic-net algorithm is known for handling high-dimensional data and selecting relevant features. The above formula, λ represents the weight coefficient of the linear regression and regularization terms, while α determines the balance between the L1 (Lasso regression) and L2 (Ridge regression) norms. For α=0 , the model is equivalent to ridge regression, and for α=1 , it becomes equivalent to lasso regression. The weight coefficients assigned to the features in the Elastic-Net model can quantify the contribution of FC pairs between different brain regions to predicting cognitive traits. To construct our brain functional decoding models, we tailored them for each specific fMRI state (as depicted in the top half of Figure 1). Initially, we screened out edges highly correlated with connectome strength. Subsequently, we employed a 10-fold cross-validation approach to creating regression models to decipher task-specific connectivity patterns. By aggregating the non-zero coefficients obtained from each fold in the Elastic-Net model, we obtained a functional subnetwork that best reflected the specificity of the given task (Caunca et al., 2021). To assess the reliability of the prediction outputs, we combined the predictors from each fold and performed a permutation test. Specifically, we calculated the Pearson correlation coefficient between the predicted and observed (random shuffled) scores. The permutation test probability was determined by evaluating the frequency of correlation coefficients in a set of 10,000 permutations that exceeded the initial coefficient.

### 2.5 Graph theory measures the connectome

Changes in graph theory measures of brain connectome have been recognized as significant aspects of various brain diseases (Savanth et al., 2022). By quantifying the graph-theoretical properties, researchers can gain insights into the essential brain regions and unravel the underlying organizational principles of the brain network (Fallahi et al., 2021; Zhang T. et al., 2021; Zamani et al., 2022). Our investigation included several graph theory measures as supplementary features for disease classification. These measures, namely graph strength, clustering coefficient, local efficiency, page rank centrality, betweenness centrality, eigenvector, flow coefficient, and k-coreness centrality, were calculated based on binary or weighted graphs after implementing a sparsity threshold (Wang B. et al., 2022). The ideal sparse brain graphs were constructed by optimizing the global brain efficiency, and the graph theory features extracted from the corresponding task-specific brain nodes.

### 2.6 Task-specific prior-knowledge graph neural network model

The population and brain parcellation methods are two commonly used GNN frameworks for diagnosing brain diseases. The population graph methodology involves constructing a graph representation at the population level (Parisot et al., 2017, 2018), while the brain-level graph methodology focuses on building graphs based on individual brain connectivity patterns (Felouat and Oukid, 2020; Wang L. et al., 2021). In our study, we employed a population GNN for further computations after decoding task-specific brain regions (as shown in the bottom half of Figure 1). We chose the population GNN approach due to its superior classification performance demonstrated in previous studies (Pan J. et al., 2022). Neuropsychological scale score, gender, or age were considered as the set of non-imaging phenotypic features $N=\left(N_{h}\right)$ . The adjacency weights of the population graph were defined as follows:

(2) $$W\left(x,y\right)=Sim\left(A_{x},A_{y}\right)\overset{H}{\underset{h=1}{\sum }}\gamma \left(N_{h}\left(x\right),N_{h}\left(y\right)\right)$$

where $Sim\left(A_{x},A_{y}\right)$ is a similarity measure between subjects x and y , γ is the distance between phenotypic measures. For every category in *h*, we adopt a threshold θ and define γ as a unit-step function:

(3) $$\gamma \left(N_{h}\left(x\right),N_{h}\left(y\right)\right)=\{\begin{array}{c} 1,if\;\left|N_{h}\left(x\right)-N_{h}\left(y\right)\right|<\theta \\ 0,\mathrm{otherwise} \end{array}\;$$

The similarity of graph features was defined as:

(4) $$Sim\left(A_{x},A_{y}\right)=\exp\left(-\frac{{\left[\rho \left(F\left(x\right),F\left(y\right)\right)\right]}^{2}}{2\sigma^{2}}\right)$$

Where ρ is the correlation distance, and σ determines the width of the kernel. Due to network connectivity and graph theory measures based on the interconnected nodes on both sides of the edges to form subnetworks, the features remain in a relatively high dimension. We adopt a ridge classifier to perform recursive feature elimination (RFE) with a fixed number of features (Ravishankar et al., 2016). In the graph convolutional component of the TSP-GNN model, the normalized graph Laplacian function of a weighted graph G=({V,E,W}) is defined as $L=I_{N}-D^{-1/2}WD^{1/2}$ where $I_{N}$ and D are, respectively, the identity matrix of size N∗N and diagonal degree matrix. The GNN architecture is derived from (Parisot et al., 2018), consists with L fully convolutional hidden layers activated using the Rectified Linear Unit (ReLU) function.

(5) $$g_{\theta }*Χ=g_{\theta }\left(L\right)Χ=g_{\theta }\left(U\Lambda U^{T}\right)Χ=Ug_{\theta }\left(\Lambda \right)\;U^{T}Χ$$

The input layer encompasses the entire population graph, while a SoftMax activation function follows the output layer. To evaluate the performance of our model, we employed a five-fold cross-validation approach across all databases. During training, the training fold consisted of a subset of tagged graph nodes, the loss function was assessed, and gradients were backpropagated on this subset.

### 2.7 Compare with other classification methods

The current research comprehensively compared the TSP-GNN method with various machine learning techniques, deep learning models, and graph neural networks. Specifically, the comparison included support vector machine (SVM), K-nearest neighbor (KNN), and several ensemble learning methods. In addition, we included two deep neural networks (DNN) methods, namely multilayer perceptron (MLP) and convolutional neural networks (CNN). The MLP method, a supervised feedforward neural network which consists of one hidden layer, was connected to the stacked autoencoder (Parisot et al., 2018). The CNN method uses the most classical design, using dropout and linear layers to achieve reduction and forecast. As for the GNN model, we employed MAGE and EV-GNN, which have demonstrated superior performance in previous studies. It is worth noting that the original MAGE utilizes a variety of brain atlas features to improve the accuracy of disease diagnosis (Wang Y. et al., 2022). We adopted this concept in our paper to effectively integrate relevant prior information from multiple task modalities. Additionally, the EV-GNN model demonstrated the ability to automatically integrate imaging data and phenotype data within a learnable adaptive population graph (Huang and Chung, 2020).

## 3 Results

### 3.1 Functional connectivity patterns of different cognitive tasks

**Figure 2:**
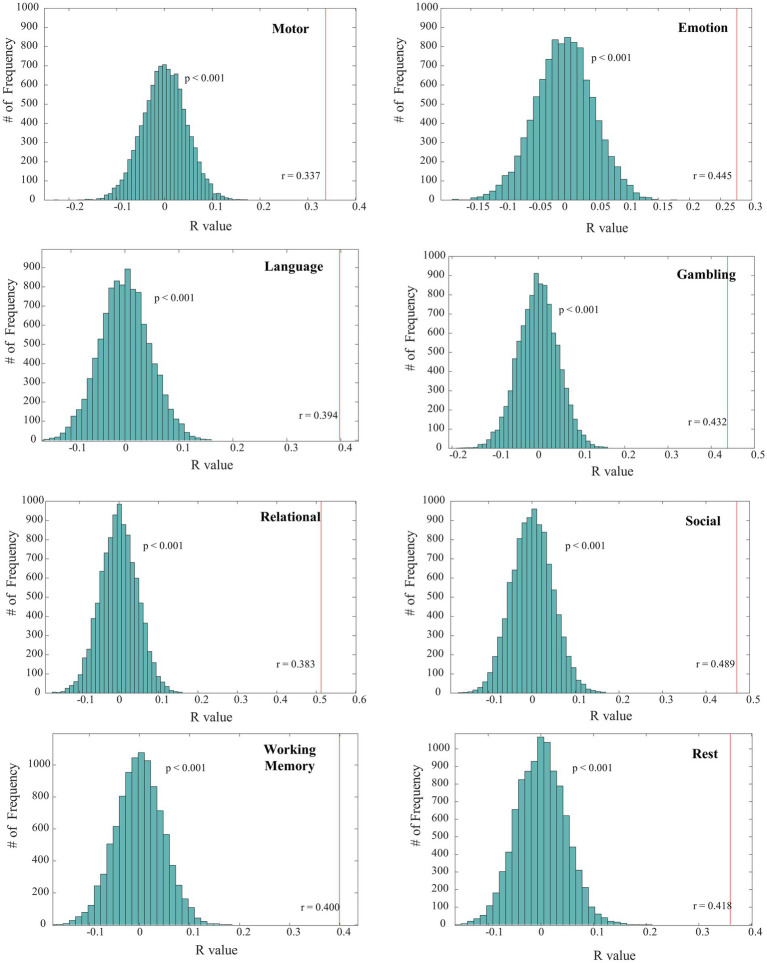
Results of permutation tests on task-state and resting-state fMRI decoding. The green histograms illustrate the correlation values’ distribution between the predicted task performances and those obtained from 10,000 permutation tests. The red line marks the correlation from the predictions of the original Elastic-Net model to the actual outcomes, clearly showing that the permutation test outcomes systematically register below the baseline correlation.

Our study demonstrates that brain connectivity patterns exhibit both task-specific characteristics and commonalities. We observed that the decoded edges traverse multiple functional brain regions and are distributed across various intrinsic resting-state networks (RSNs), indicating shared patterns across different tasks. The assessment metrics presented in Table 2 indicate the strength of the decoding results, with all expected correlation coefficients (r values) exceeding 0.3 and the corresponding value of ps being less than 0.05. Notably, we found that the prediction models for all tasks passed the permutation test, confirming the reliability and consistency of our decoding results (Figure 2). In addition to the permutation test, we employed several evaluation measures to assess the performance of the decoding models. These measures included the mean squared error (MSE), explained variance score (EVS), and mean absolute error (MAE). By examining these metrics, we gained further insights into the accuracy and precision of our prediction models.

**Table 2:** Prediction and evaluations of various cognitive abilities.

Task	*r* value	Value of *p*	*R*_2_	MSE	EVS	MAE
W	0.400	0.017*	0.125	0.851	0.141	0.730
S	0.489	0.044*	0.205	0.741	0.228	0.685
L	0.394	0.019*	0.147	0.845	0.151	0.747
E	0.445	0.018*	0.169	0.803	0.186	0.725
R	0.383	0.016*	0.112	0.873	0.141	0.734
M	0.337	0.043*	0.097	0.887	0.108	0.713
G	0.432	0.006**	0.165	0.816	0.183	0.740
REST	0.418	0.011*	0.149	0.824	0.161	0.715

W, working memory; E, emotion processing; L, language; S, social cognitive; R, relation processing; M, motor; G, gambling.

### 3.2 Anatomical and functional localization of task-specific network edges

**Figure 3:**
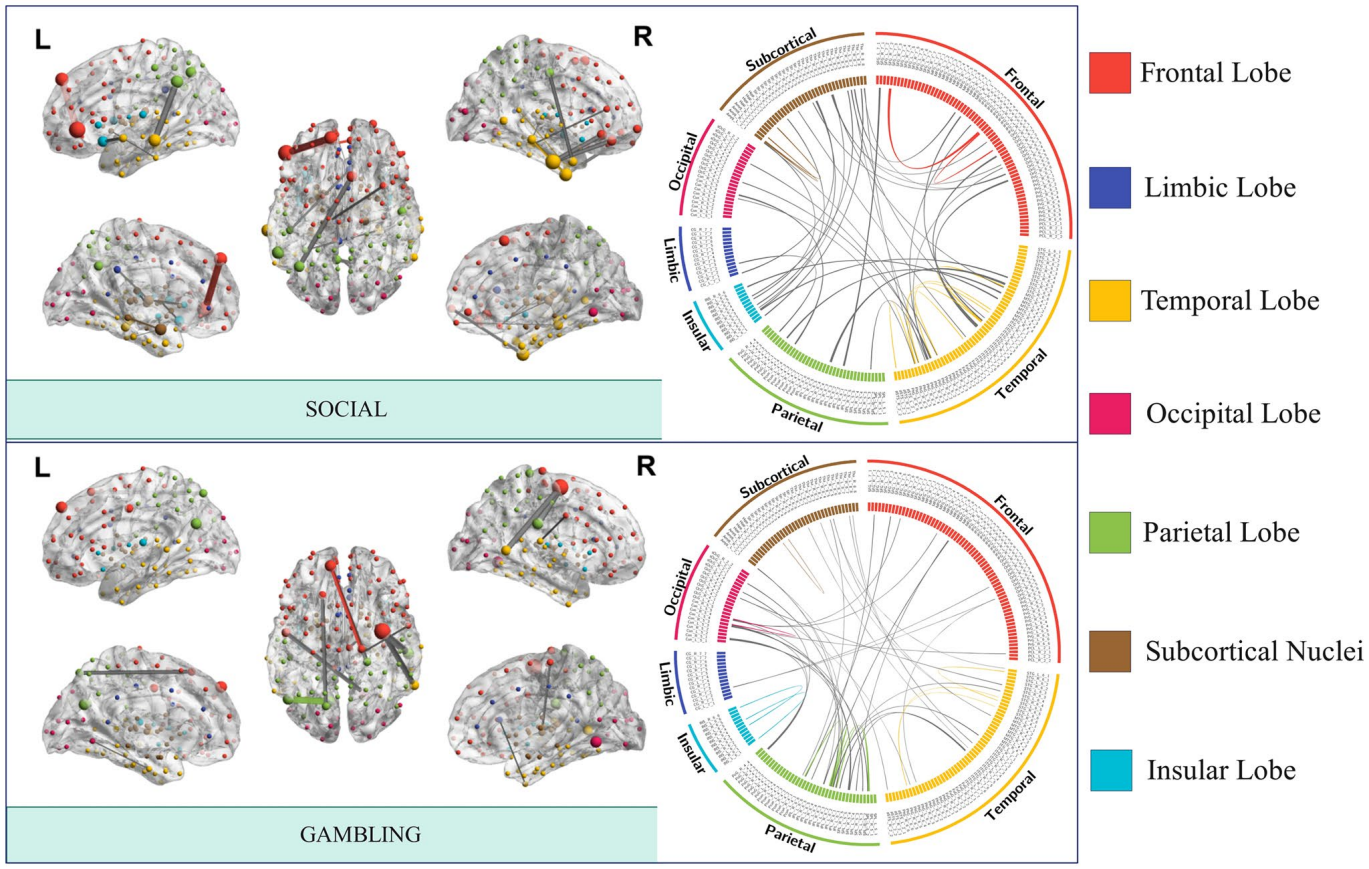
FCs with the best task performance prediction capability. The nodes and edges of the brain network are created by averaging the FC strength of a particular task across all people, and the strength determines the node size and edge thickness. Connections within a module are depicted using the same color as the module in which it is situated, whereas gray lines represent inter-module connections.

Significant interconnections were identified by analyzing the non-zero coefficients in the Elastic-Net model. Our analysis results revealed the most prominent interconnections associated with each task state, with the following number of edges identified: emotion (47 edges), gambling (46 edges), language (21 edges), motor task (15 edges), relational (27 edges), social (44 edges), working memory (22 edges), and rest (99 edges). Importantly, it was observed that the seven task-specific regions were widely distributed across different anatomical locations, and the number of specific edges involved in rest-state fMRI was greater than that in task fMRI. A circular diagram has depicted the distribution of the essential connected edges of social cognition and gambling tasks (Figure 3). The specific connectivity distribution patterns of the brain networks for the other five tasks and resting-state fMRI are presented in Supplementary Figures S1, S2.

**Figure 4:**
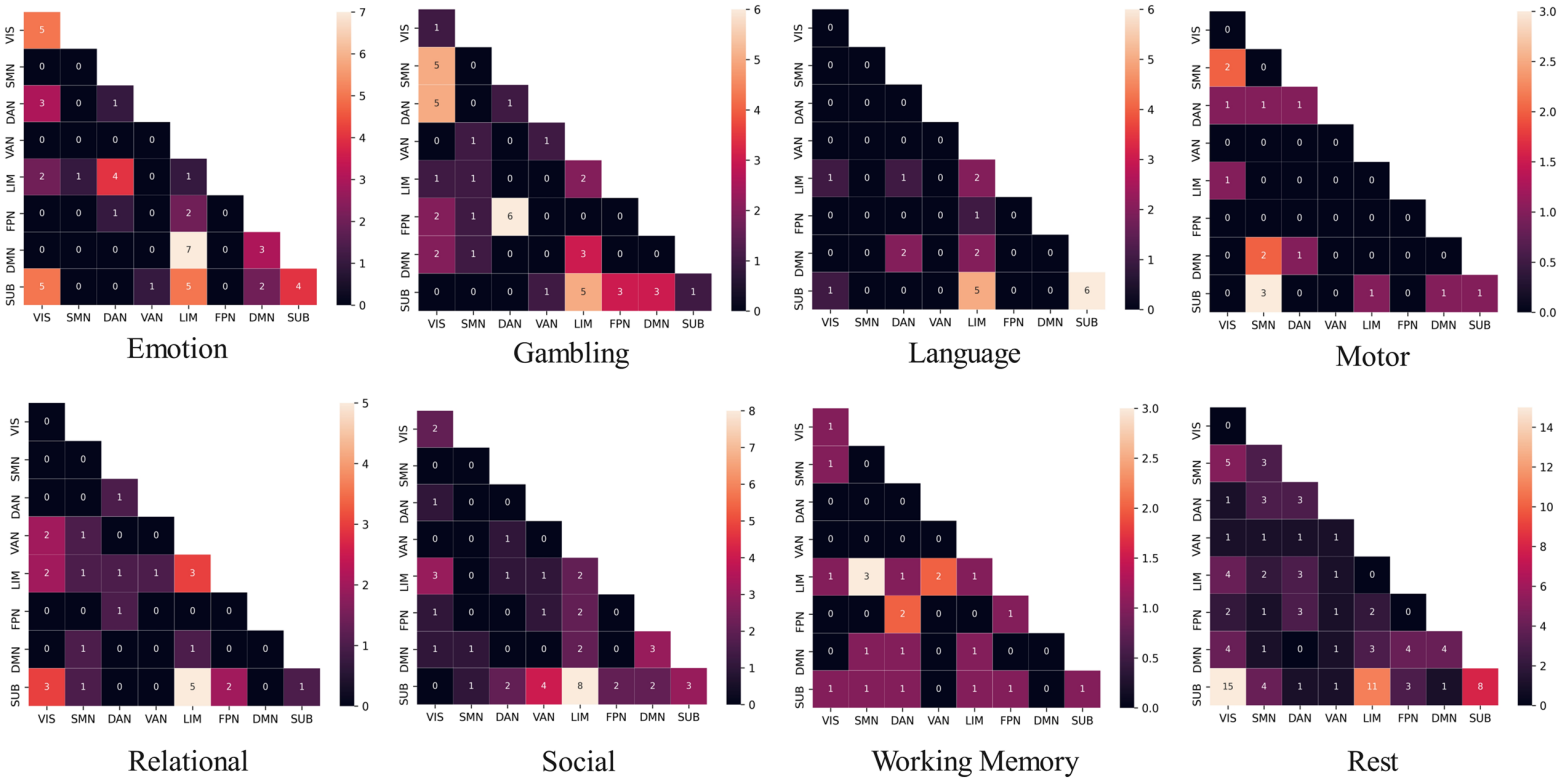
The distribution of functional brain networks associated with edges differs across decoding modes of task states. DAN, dorsal attention network; DMN, default mode network; FPN, frontoparietal network; LIM, limbic network; SMN, somatomotor network; SUB, subcortical network; VAN, ventral attention network; VIS, visual network.

The social task-related FC patterns were distributed inter-LIM-VIS, LIM-SUB, VAN-SUB networks, and intra-DMN and SUB networks. In the gambling task, participants were asked to guess the number of a mystery card. Decoding results showed significant ROIs, such as inter-insular subsystem, angular gyrus (IPL_L_6_2), supramarginal gyrus (IPL_L_6_3), superior parietal lobule (SPL), precuneus (Pcun_L_4_1), right cuneus (Cun_R_5_3, Cun_R_5_4). From the RSN perspective, brain edges related to gambling or risk decision were mainly distributed inter- SMN-VIS, DAN-VIS, DAN-FPN, and LIM-SUB networks (Figure 4), indicating a broader cross-network interaction. In the resting state, the FC pattern has the highest number of brain edges and almost exhibits the highest proportion of connections within brain anatomical locations. Resting-state fMRI predicts general intelligence, which includes reasoning, problem-solving, abstract thinking, planning, and learning, in our model.

### 3.3 Task-specific brain connectivity for disease classification

To evaluate the impact of task-specific prior knowledge on brain disease classification, we extracted a subnetwork comprising all the nodes involved in the task-based connectome. Additionally, we incorporated graph-theoretical properties of these task-specific nodes derived from binary and weighted brain network analyzes. These steps allowed us to amalgamate FC strength with graph metrics, culminating in a refined set of input features for the GNN model. This particular methodology facilitated a comprehensive exploration of the influence of prior knowledge on disease categorization. Notably, demographics and behavioral statistics are also incorporated into the construction of the population graph. Table 3 presents the classification performance ranking by task paradigm of each dataset. The findings suggest that the classification of different types of mental illnesses exhibited a preference for the specific task prior knowledge. As two prevalent neurodevelopmental disorders, ASD and ADHD frequently co-occur. Interestingly, they exhibited distinct task preferences in classification tasks. For ADHD, task-specific features related to social and relational processing tasks can achieve higher classification accuracy. In contrast, the ABIDE dataset has shown that gambling, motor, and relational processing are the top three task-specific patterns that yielded the best classification performance.

**Table 3:** The implications of priori information decoded by different tasks on neuropsychiatric disease classification.

ADHD			ABIDE		
TASK	AUC	ACC	TASK	AUC	ACC
M	0.697	0.653	S	0.670	0.652
W	0.700	0.653	REST	0.696	0.655
L	0.705	0.632	W	0.723	0.702
G	0.705	0.636	E	0.724	0.680
REST	0.705	0.649	L	0.728	0.722
E	0.705	0.658	R	0.734	0.688
R	0.711	0.680	M	0.739	0.711
S	0.720	0.651	G	0.760	0.670

### 3.4 Investigate the categorization effect of various task combination models

We further conducted task-specific prior knowledge experiments on disease classification to evaluate previous task information’s influence on disease classification and investigate if information complementarity between tasks may enhance diagnosis performance. We selected four, five, and six tasks from seven different task categories to create diverse combinations, $C_{7}^{4},C_{7}^{5}\;\mathrm{and}\;C_{7}^{6}$ . We presented the top three ranking AUC results for each combination of task quantities, as shown in Table 4. Our findings reveal that $C_{7}^{4}$ yields the best classification performance, whereas increasing the accuracy of $C_{7}^{5}\;\mathrm{and}\;C_{7}^{6}$ .

**Table 4:** The effects of task decoding information combination patterns on neuropsychiatric disease classification.

	ADHD			ABIDE		
	TASK group	AUC	ACC	TASK group	AUC	ACC
Task_4	E_M_S_W	0.722	0.666	G_L_R_W	0.740	0.691
	E_M_R_S	0.723	0.660	G_M_R_W	0.754	0.705
	M_R_S_W	0.724	0.671	E_G_S_W	0.759	0.702
Task_5	E_G_L_R_S	0.721	0.669	G_L_M_R_W	0.738	0.716
	L_M_R_S_W	0.721	0.662	E_G_R_S_W	0.740	0.670
	E_G_M_R_S	0.721	0.656	E_G_L_M_S	0.741	0.705
Task_6	E_L_M_R_S_W	0.712	0.662	E_G_M_R_S_W	0.722	0.680
	E_G_L_M_R_S	0.715	0.656	E_G_L_M_R_S	0.725	0.677
	G_L_M_R_S_W	0.720	0.680	E_G_L_M_S_W	0.728	0.694
Task_7	G_L_M_R_S_W_E	0.712	0.659	G_L_M_R_S_W_E	0.732	0.677

From the perspective of the classification effect of the combination mode, brain diseases exhibit differential task combination preferences. Specifically, the combination of M_R_S_W achieved the best classification results on the ADHD dataset. Not exactly consistently, the combination of E_G_S_W performed best on the ABIDE dataset. Compared with single-task experiments, the classification performance is slightly improved by selecting task-specific information for combinations. Additionally, the types of tasks frequently appearing in the 4-task combination also perform well in single-task experiments.

**Figure 5:**
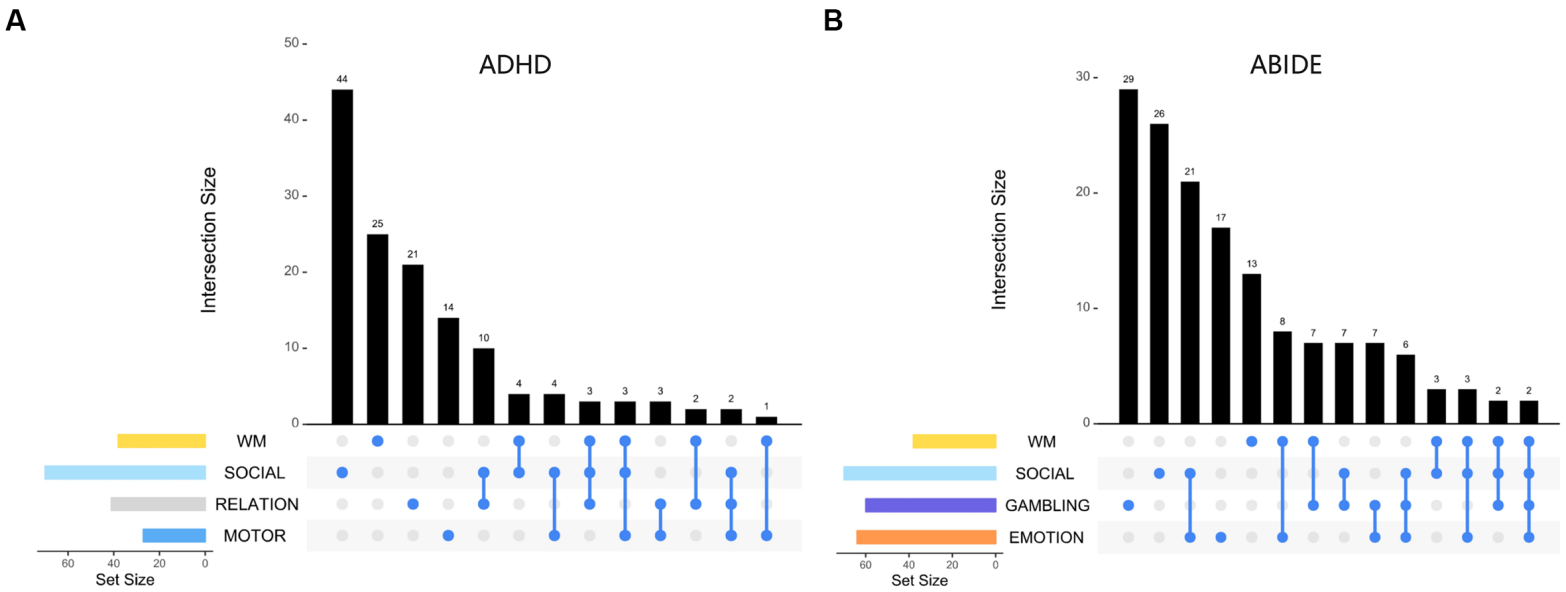
Visualizing the distribution set of nodes involved in the optimal task combination: **(A)** for ADHD dataset, **(B)** for ABIDE dataset. The findings indicate that the decoded results (node distribution) are relatively independent, with a low proportion of nodes belonging to the intersection of multiple tasks.

We displayed the task-specific brain node interactions effect for best task combinations under ADHD and ABIDE datasets (Figure 5). The best task combinations for these two diseases involve working memory and social cognition. In ADHD, social cognition and working memory tasks contribute the most nodes, whereas gambling and social cognition do in ABIDE. Table 5 shows that when all ROIs are included, i.e., FC features (246 * 245/2 = 30,135) or graph theory features (15 attributes, 246 * 15 = 3,690), the classification accuracy decreases, further highlighting the superiority of task-specific nodes.

**Table 5:** Comparing the classification performance of task-specific features and whole brain features during two datasets.

	ADHD		ABIDE	
	AUC	ACC	AUC	ACC
All FCs	0.706	0.649	0.693	0.671
All Graph Measures	0.675	0.630	0.738	0.719
FCs + Graph Measures	0.663	0.621	0.739	0.716
Best TSP-GNN	0.724	0.671	0.759	0.702

### 3.5 Comparison results with other baseline models

**Figure 6:**
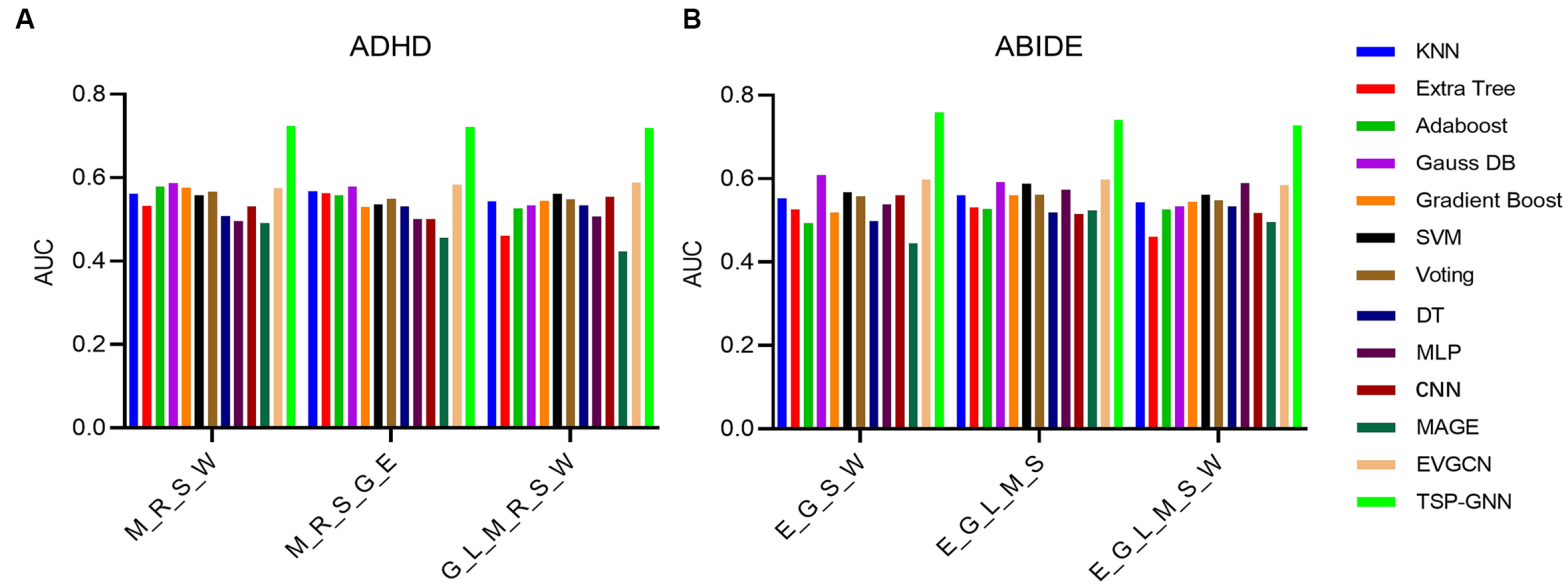
On the ADHD **(A)** and ABIDE **(B)** datasets, the classification performance of the TSP-GNN framework was compared to that of various machine learning and deep learning methods. The task priors used were combinations of the best four, five, and six combinations described in section 3.3. M_R_S_W stands for a task combination of motor, relational processing, social cognitive, and working memory tasks, and the remaining acronyms are similar.

In this present investigation, various machine learning and deep learning methods were used to illustrate the superiority of the TSP-GNN model in ND diagnosis. To ensure the uniformity of input features, we conducted experiments using the optimal task combination stated in section 3.4. In the classification experiments of ADHD and ABIDE datasets, TSP-GNN has obtained the optimal results (Figure 6), and the detailed numerical values of the classification results can be found in Supplementary Tables S2, S3. In comparison to classic machine learning approaches such as SVM (Abraham et al., 2017) and ensemble learning (Liu et al., 2020), GNN models the individual-based topologies structure (Zhou and Zhang, 2021) between subjects utilizing participant similarity, which is advantageous for enhancing classification performance. After numerous layers of graph convolution computation, highly relevant characteristics are continually aggregated (Wang L. et al., 2021). MLP and CNN apply fully-connected and convolutional layers to achieve dimensionality reduction on brain network features, which are spatial topological graphs between brain areas and cannot be equated to the image receptive field (Dvornek et al., 2017; Khosla et al., 2018). The TSP-GNN architecture blends multi-task information from FC characteristics and graph measures to collect better and characterize the most discriminative information than typical machine learning and deep neural network models.

## 4 Discussions

This study represents the first investigation in brain disease classification that focuses explicitly on task-specific FC patterns. Task-based fMRI offers distinct advantages in exploring and understanding the mechanisms and brain-behavior relationships specific to cognitive impairments, which may not be evident in resting-state fMRI. Task paradigms provide structured cognitive engagement (Jiang et al., 2020; Yoo et al., 2022), allowing for a better examination of individual differences in critical neural circuits (Greene et al., 2018). Given the advantages of task fMRI, we employed the Elastic-Net regression model to explore task-specific FC patterns decoded relying on brain-behavior relationships. Additionally, we used resting-state fMRI to decode general intelligence as a baseline for comparison with task-specific FC (Dubois et al., 2018; Thiele et al., 2022; Anderson and Barbey, 2023). The decoding results for different tasks exhibited high heterogeneity, highlighting the brain regions and connectivity patterns that are more representative of the current task, in contrast to traditional supervised models based on task labels alone (Zhang et al., 2022).

In decoding brain-behavior relationships, selecting predictors and outcomes for the predictive model is a topic worthy of exploration. While predictions about various behaviors can be made based on resting-state data, our research prioritizes focus on the relationship between task-state fMRI and corresponding cognitive performance under task scenarios. The predictive modeling based on task-state fMRI is inspired by the potential of task-state FC to enhance cognitive outcome prediction (Jiang et al., 2020). Additionally, it fully explores the multiple task states within the HCP dataset. We consider utilizing the Acc/RT ratio as a behavioral index for tasks with accuracy and response speed metrics in predicting behavioral performance. Literature also conceptualizes the trade-off between speed and accuracy as ‘throughput’ (Thorne, 2006; Heitz, 2014). It reflects the accuracy of the response and its rapidity, thereby providing a composite measure of cognitive processing efficiency.

Our research corroborates the efficacy of integrating task-specific connectome priors into classification models for diagnosing a spectrum of psychiatric disorders across various datasets. Specifically, enhanced classification performance is observed in differentiating diseases when utilizing FC patterns associated with specific cognitive domains (Chauvin et al., 2021). Network patterns related to working memory tasks contribute significantly to both ADHD and ASD datasets. The previous study also reveals that impairments in working memory are prevalent across psychiatric conditions (Wang X. L. et al., 2021), and memory assessments are crucial for predicting and mitigating high-risk disorders (Seabury and Cannon, 2020). In classifying ADHD, leading tasks also encompass motor task, social cognition, and relational processing. Previous studies have demonstrated that severe declines in social cognition and motor speed (Haining et al., 2020) correlate with a high risk of clinical psychiatric conditions. ADHD is also associated with abnormalities in the large-scale cognitive control network that impact social attention (Fateh et al., 2022), with adolescents among the patient population exhibiting impairments in social cognition and communication abilities (Chen and Chen, 2020). Children with ADHD have a deficit in relational reasoning (Brunamonti et al., 2017), a skill subtending the acquisition of many cognitive abilities and social rules. In the classification of the ABIDE dataset, leading tasks also encompass emotion, gambling, and social cognition. Facial emotion recognition disorder is typical of people with autism. Facial emotion recognition disorder is a classic symptom of autism (Yeung, 2022). Cognitive inflexibility in people with autism appears characterized by the unwillingness to switch toward processing socio-emotional information (Latinus et al., 2019). Individuals with ASD frequently report difficulty making flexible decisions across various contexts to resolve social or moral conflicts(Tei et al., 2022). Concurrently, studies based on gambling paradigms also suggest they tend to exhibit a more cautious decision-making style (Hosozawa et al., 2021).

Integrating multi-task FC and graph theory has further enhanced classification accuracy, achieving optimal performance using four task combinations. However, the addition of features from more tasks did not continue to improve classification results, presenting an intriguing avenue for investigation. In constructing brain FC-based diagnostic models, selecting features is more critical than quantity (Du et al., 2018; Chen et al., 2020). An increased number of features may offer a richer representation of task-specific FC information, but it can also lead to the “curse of dimensionality”—a phenomenon where the introduction of noise, overfitting, and the increased difficulty of identifying meaningful patterns in high-dimensional spaces may decrease classification performance (Wee et al., 2014; Barbieri et al., 2022). Our research also validates that opting for a more suitable selection of features, rather than simply increasing their number, is the superior strategy.

The TSP-GNN system achieves a balanced trade-off between model interpretability and classification performance. In contrast to previous studies that incorporated whole-brain connectome features, our model utilizes a task-specific FC pattern, which enhances the interpretability of features by linking them to specific cognitive activities. Furthermore, the classification stage of the TSP-GNN framework employs a population graph model, simplifying the modeling of brain areas as nodes and improving classification performance. Regarding classification performance, our TSP-GNN outperforms various classical machine learning and deep network models, underscoring the superiority of our task-prioritized population graph model in detecting brain diseases. Although our classification accuracy may differ from recent studies (Chen et al., 2021; Pan J. et al., 2022), this may be due to trade-offs and parameter adjustments made during model construction. Our framework prioritizes the interpretation of cognitive processes and their extended values related to underlying disease, and task-specific prior information from brain areas can be easily transferred to other studies of cognitive brain disease and disorders. In summary, we consider the decoding model in our TSP-GNN framework as a pre-task, effectively reducing feature dimensionality and elucidating the role of task-specific prior information in the classification model for brain disease diagnosis. The model effectively bridges the gap between cognitive behavior decoding and brain illness research, offering valuable insights and serving as a reference for task-related investigations in brain diseases.

Several considerations need to be addressed in our research. Firstly, it should be acknowledged that the ADHD and ABIDE illness cohorts in our study were not comprehensive and may not represent all available data sources. The inherent imbalance resulting from variations in data collection parameters and equipment across different locations is a significant challenge in our investigation. Constrained by the differing intended uses of data acquisition between HCP and ND, a strict age match between groups was not feasible, thus warranting further investigation into the exclusion of age-related differences in brain network impacts (Zhang et al., 2023). Secondly, the task-specific FC derived from the regression process has enhanced the efficacy of disease diagnosis and is considered, to some extent, correlative rather than causally direct. Employing causal correlation-based FC (Sanchez-Romero et al., 2023) and evidence of neural modulation (Zhou et al., 2020) based on brain networks holds promise for overcoming this limitation. Lastly, our current classification results can be further enhanced by refining the incorporation of prior information and optimizing future models to approach state-of-the-art performance. Continual efforts to improve the quality of prior knowledge and refine model development are necessary to ensure our approach remains at the forefront of research in this field.

Future research aims to develop deep learning models integrating cognitive performance and task state labels for brain decoding. Recognizing the intricate relationship between brain decoding and classification, despite their distinct objectives, we intend to explore the application of zero-shot learning and advanced transfer learning models that can achieve mutual benefits for both brain function decoding and disease classification tasks (Zhang P. et al., 2021). An exciting prospect is the collection of psychiatric disorder data using appropriate task paradigms in clinical settings (Birba et al., 2022). By incorporating task performance in actual clinical circumstances, we can investigate and evaluate the underlying causes of illnesses, expand our prior knowledge about task-based brain activity, and further optimize our models accordingly. Our future endeavors aim to bridge the gap between brain decoding and disease classification by developing advanced deep-learning models informed by clinical data and task performance. This approach has the potential to significantly contribute to the field by providing valuable insights into the underlying mechanisms of brain disorders and facilitating more accurate diagnoses.

## 5 Conclusion

The present study introduces a novel TSP-GNN framework to improve brain disease classification. By leveraging functional connection-based cognitive performance prediction, this study decodes task-specific FC patterns and transfers them as prior knowledge for diagnosing ND. As far as we know, this study represents the first attempt to transfer task-specific connectivity patterns as a priori knowledge in brain disease research. Our results demonstrate that integrating task-specific priors leads to improved classification accuracy compared to traditional methods. The finding highlights the informativeness of task-specific connection patterns. Besides, the optimal task combinations for each kind of ND offer valuable insights into the underlying mechanisms of that brain disease. By incorporating task-specific connectivity patterns, our framework enhances the understanding and prediction of brain diseases, opening up new avenues for future investigations in this domain.

## Data availability statement

The original contributions presented in the study are included in the article/Supplementary material, further inquiries can be directed to the corresponding authors.

## Author contributions

JL: Conceptualization, Investigation, Methodology, Software, Writing – original draft, Writing – review & editing. L-ZY: Conceptualization, Methodology, Supervision, Writing – review & editing. HL: Conceptualization, Supervision, Writing – review & editing.

## Footnotes

^1^https://preprocessed-connectomes-project.org/adhd200/

^2^https://fcon_1000.projects.nitrc.org/indi/abide/abide_I.html
