# Supplementary material for: TSP-GNN: a novel neuropsychiatric disorder classification framework based on task-specific prior knowledge and graph neural network
Source: Front Neurosci. 2023 Dec 21;17:1288882. doi: 10.3389/fnins.2023.1288882 (PMC10768162; doi:10.3389/fnins.2023.1288882)
Supplement: Supplementary file 1 [file Data_Sheet_1.docx]

Supplementary Material

TSP-GNN: A novel neuropsychiatric disorder classification framework based on task-specific prior knowledge and graph neural network

Jinwei Lang^1,2^, Li-Zhuang Yang^1,3*^, Hai Li ^1,3*^

*** Corresponding Author:**

lzyang@cmpt.ac.cn; hli@cmpt.ac.cn

# Supplementary Tables

Supplementary Table 1. A brief description of each HCP cognitive condition.

| **Task** | **Frames/ run** | **Duration**  **(min: sec)** | **Description** | |
| --- | --- | --- | --- | --- |
| **Emotion processing** | 176 | 2:16 | The task involves participants deciding which of two faces or shapes matches the one at the top of the screen (Manuck et al., 2007). The faces have either an angry or fearful expression and are presented in blocks of 6 trials. Each block is preceded by a task cue, with three face blocks and three shape blocks in each run. |  |
| **Gambling** | 253 | 3:12 | Participants play a card guessing game where they must guess the number on a mystery card to win or lose money (Delgado et al., 2000). The potential card numbers range from 1-9, and participants indicate if the number is more or less than 5. Feedback is given for each trial, and the task is presented in blocks of 8 trials, mainly reward or loss. The task is presented in two runs with a fixed block in the middle. |  |
| **Language** | 316 | 3:57 | The task consists of two runs, each with four blocks of a story task and four blocks of a math task, with the math trials matching the length of the story task (Binder et al., 2011). The story blocks present auditory stories followed by a question, while the math task presents addition and subtraction problems with two choices. The task is adaptive to maintain a similar level of difficulty for participants. |  |
| **Motor** | 284 | 3:34 | The task requires participants to respond to visual cues by tapping their fingers, squeezing their toes, or moving their tongues to map motor areas (Buckner et al., 2011). Each movement block lasts 12 seconds, with two runs consisting of 13 blocks each. There are also three 15-second fixation blocks per run. |  |
| **Relational processing** | 232 | 2:56 | The task involves participants being presented with six different shapes filled with 1 of 6 different textures (Smith et al., 2007). In the relational processing condition, participants are presented with two pairs of objects and are asked to decide what dimension differs across the top pair of objects and then whether the bottom pair of objects also differ along that same dimension. In the control matching condition, participants are shown two objects at the top of the screen and one object at the bottom of the screen, and a word in the middle of the screen. The task lasts for 18 seconds per block, and there are three relational blocks, three matching blocks, and three 16-second fixation blocks. |  |
| **Social cognition** | 274 | 3:27 | Participants watched 20-s video clips of shapes interacting or moving randomly and had to judge whether there was a mental interaction between the shapes, no interaction, or they were unsure(Castelli et al., 2002; White et al., 2011). Two groups created the videos, and the task had two runs with 5 video blocks each and 5 fixation blocks. |  |
| **Working memory** | 405 | 5:01 | Participants completed a task that combined the category-specific representation and working memory tasks (Gevins and Cutillo, 1993). They viewed pictures of places, tools, faces, and body parts and performed a 2-back or 0-back working memory task on separate blocks. Each run consisted of 8 task blocks and 4 fixation blocks, with a 2.5s cue indicating the task type at the start of each block. The stimulus was presented for 2 seconds with a 500 ms inter-task interval. |  |
| **Rest** | 1200 | 14:33 | Four runs of resting-state fMRI data were collected, each approximately 15 minutes long, with alternating phase encoding directions. Participants were instructed to keep their eyes open and relaxed, fixating on a bright crosshair presented in a dark room. |  |

Supplementary Table 2. Anatomy and functional network division of Human Brainnetome Atlas.

| Index | name | region | Network | MNI | Lobel | Index | name | region | Network | MNI | Lobel |
| --- | --- | --- | --- | --- | --- | --- | --- | --- | --- | --- | --- |
| 1 | A8m | SFG_L_7_1 | 6 | -5,15,54 | Frontal | **124** | cpSTS | pSTS_R_2_2 | 4 | 57,-40,12 | Temporal |
| 2 | A8m | SFG_R_7_1 | 4 | 7,16,54 | Frontal | **125** | A7r | SPL_L_5_1 | 3 | -16,-60,63 | Parietal |
| 3 | A8dl | SFG_L_7_2 | 7 | -18,24,53 | Frontal | **126** | A7r | SPL_R_5_1 | 3 | 19,-57,65 | Parietal |
| 4 | A8dl | SFG_R_7_2 | 6 | 22,26,51 | Frontal | **127** | A7c | SPL_L_5_2 | 3 | -15,-71,52 | Parietal |
| 5 | A9l | SFG_L_7_3 | 7 | -11,49,40 | Frontal | **128** | A7c | SPL_R_5_2 | 3 | 19,-69,54 | Parietal |
| 6 | A9l | SFG_R_7_3 | 7 | 13,48,40 | Frontal | **129** | A5l | SPL_L_5_3 | 3 | -33,-47,50 | Parietal |
| 7 | A6dl | SFG_L_7_4 | 3 | -18,-1,65 | Frontal | **130** | A5l | SPL_R_5_3 | 3 | 35,-42,54 | Parietal |
| 8 | A6dl | SFG_R_7_4 | 3 | 20,4,64 | Frontal | **131** | A7pc | SPL_L_5_4 | 2 | -22,-47,65 | Parietal |
| 9 | A6m | SFG_L_7_5 | 2 | -6,-5,58 | Frontal | **132** | A7pc | SPL_R_5_4 | 2 | 23,-43,67 | Parietal |
| 10 | A6m | SFG_R_7_5 | 2 | 7,-4,60 | Frontal | **133** | A7ip | SPL_L_5_5 | 3 | -27,-59,54 | Parietal |
| 11 | A9m | SFG_L_7_6 | 7 | -5,36,38 | Frontal | **134** | A7ip | SPL_R_5_5 | 3 | 31,-54,53 | Parietal |
| 12 | A9m | SFG_R_7_6 | 6 | 6,38,35 | Frontal | **135** | A39c | IPL_L_6_1 | 1 | -34,-80,29 | Parietal |
| 13 | A10m | SFG_L_7_7 | 7 | -8,56,15 | Frontal | **136** | A39c | IPL_R_6_1 | 1 | 45,-71,20 | Parietal |
| 14 | A10m | SFG_R_7_7 | 7 | 8,58,13 | Frontal | **137** | A39rd | IPL_L_6_2 | 6 | -38,-61,46 | Parietal |
| 15 | A9/46d | MFG_L_7_1 | 4 | -27,43,31 | Frontal | **138** | A39rd | IPL_R_6_2 | 6 | 39,-65,44 | Parietal |
| 16 | A9/46d | MFG_R_7_1 | 6 | 30,37,36 | Frontal | **139** | A40rd | IPL_L_6_3 | 3 | -51,-33,42 | Parietal |
| 17 | IFJ | MFG_L_7_2 | 6 | -42,13,36 | Frontal | **140** | A40rd | IPL_R_6_3 | 3 | 47,-35,45 | Parietal |
| 18 | IFJ | MFG_R_7_2 | 6 | 42,11,39 | Frontal | **141** | A40c | IPL_L_6_4 | 7 | -56,-49,38 | Parietal |
| 19 | A46 | MFG_L_7_3 | 6 | -28,56,12 | Frontal | **142** | A40c | IPL_R_6_4 | 6 | 57,-44,38 | Parietal |
| 20 | A46 | MFG_R_7_3 | 6 | 28,55,17 | Frontal | **143** | A39rv | IPL_L_6_5 | 3 | -47,-65,26 | Parietal |
| 21 | A9/46v | MFG_L_7_4 | 6 | -41,41,16 | Frontal | **144** | A39rv | IPL_R_6_5 | 7 | 53,-54,25 | Parietal |
| 22 | A9/46v | MFG_R_7_4 | 6 | 42,44,14 | Frontal | **145** | A40rv | IPL_L_6_6 | 2 | -53,-31,23 | Parietal |
| 23 | A8vl | MFG_L_7_5 | 7 | -33,23,45 | Frontal | **146** | A40rv | IPL_R_6_6 | 2 | 55,-26,26 | Parietal |
| 24 | A8vl | MFG_R_7_5 | 6 | 42,27,39 | Frontal | **147** | A7m | Pcun_L_4_1 | 6 | -5,-63,51 | Parietal |
| 23 | A6vl | MFG_L_7_6 | 3 | -32,4,55 | Frontal | **148** | A7m | Pcun_R_4_1 | 6 | 6,-65,51 | Parietal |
| 26 | A6vl | MFG_R_7_6 | 3 | 34,8,54 | Frontal | **149** | A5m | Pcun_L_4_2 | 2 | -8,-47,57 | Parietal |
| 27 | A10l | MFG_L_7_7 | 5 | -26,60,-6 | Frontal | **150** | A5m | Pcun_R_4_2 | 3 | 7,-47,58 | Parietal |
| 28 | A10l | MFG_R_7_7 | 6 | 25,61,-4 | Frontal | **151** | dmPOS | Pcun_L_4_3 | 1 | -12,-67,25 | Parietal |
| 29 | A44d | IFG_L_6_1 | 6 | -46,13,24 | Frontal | **152** | dmPOS | Pcun_R_4_3 | 1 | 16,-64,25 | Parietal |
| 30 | A44d | IFG_R_6_1 | 3 | 45,16,25 | Frontal | **153** | A31 | Pcun_L_4_4 | 7 | -6,-55,34 | Parietal |
| 31 | IFS | IFG_L_6_2 | 6 | -47,32,14 | Frontal | **154** | A31 | Pcun_R_4_4 | 7 | 6,-54,35 | Parietal |
| 32 | IFS | IFG_R_6_2 | 6 | 48,35,13 | Frontal | **155** | A1/2/3ulhf | PoG_L_4_1 | 2 | -50,-16,43 | Parietal |
| 33 | A45c | IFG_L_6_3 | 7 | -53,23,11 | Frontal | **156** | A1/2/3ulhf | PoG_R_4_1 | 2 | 50,-14,44 | Parietal |
| 34 | A45c | IFG_R_6_3 | 7 | 54,24,12 | Frontal | **157** | A1/2/3tonIa | PoG_L_4_2 | 2 | -56,-14,16 | Parietal |
| 35 | A45r | IFG_L_6_4 | 7 | -49,36,-3 | Frontal | **158** | A1/2/3tonIa | PoG_R_4_2 | 2 | 56,-10,15 | Parietal |
| 36 | A45r | IFG_R_6_4 | 6 | 51,36,-1 | Frontal | **159** | A2 | PoG_L_4_3 | 3 | -46,-30,50 | Parietal |
| 37 | A44op | IFG_L_6_5 | 4 | -39,23,4 | Frontal | **160** | A2 | PoG_R_4_3 | 2 | 48,-24,48 | Parietal |
| 38 | A44op | IFG_R_6_5 | 4 | 42,22,3 | Frontal | **161** | A1/2/3tru | PoG_L_4_4 | 2 | -21,-35,68 | Parietal |
| 39 | A44v | IFG_L_6_6 | 4 | -52,13,6 | Frontal | **162** | A1/2/3tru | PoG_R_4_4 | 2 | 20,-33,69 | Parietal |
| 40 | A44v | IFG_R_6_6 | 4 | 54,14,11 | Frontal | **163** | G | INS_L_6_1 | 2 | -36,-20,10 | Insular |
| 41 | A14m | OrG_L_6_1 | 7 | -7,54,-7 | Frontal | **164** | G | INS_R_6_1 | 2 | 37,-18,8 | Insular |
| 42 | A14m | OrG_R_6_1 | 7 | 6,47,-7 | Frontal | **165** | vIa | INS_L_6_2 | 8 | -32,14,-13 | Insular |
| 43 | A12/47o | OrG_L_6_2 | 7 | -36,33,-16 | Frontal | **166** | vIa | INS_R_6_2 | 6 | 33,14,-13 | Insular |
| 44 | A12/47o | OrG_R_6_2 | 7 | 40,39,-14 | Frontal | **167** | dIa | INS_L_6_3 | 4 | -34,18,1 | Insular |
| 45 | A11l | OrG_L_6_3 | 5 | -23,38,-18 | Frontal | **168** | dIa | INS_R_6_3 | 4 | 36,18,1 | Insular |
| 46 | A11l | OrG_R_6_3 | 6 | 23,36,-18 | Frontal | **169** | vId/vIg | INS_L_6_4 | 4 | -38,-4,-9 | Insular |
| 47 | A11m | OrG_L_6_4 | 5 | -6,52,-19 | Frontal | **170** | vId/vIg | INS_R_6_4 | 4 | 39,-2,-9 | Insular |
| 48 | A11m | OrG_R_6_4 | 5 | 6,57,-16 | Frontal | **171** | dIg | INS_L_6_5 | 2 | -38,-8,8 | Insular |
| 49 | A13 | OrG_L_6_5 | 5 | -10,18,-19 | Frontal | **172** | dIg | INS_R_6_5 | 2 | 39,-7,8 | Insular |
| 50 | A13 | OrG_R_6_5 | 5 | 9,20,-19 | Frontal | **173** | dId | INS_L_6_6 | 4 | -38,5,5 | Insular |
| 51 | A12/47l | OrG_L_6_6 | 7 | -41,32,-9 | Frontal | **174** | dId | INS_R_6_6 | 4 | 38,5,5 | Insular |
| 52 | A12/47l | OrG_R_6_6 | 7 | 42,31,-9 | Frontal | **175** | A23d | CG_L_7_1 | 7 | -4,-39,31 | Limbic |
| 53 | A4hf | PrG_L_6_1 | 2 | -49,-8,39 | Frontal | **176** | A23d | CG_R_7_1 | 7 | 4,-37,32 | Limbic |
| 54 | A4hf | PrG_R_6_1 | 2 | 55,-2,33 | Frontal | **177** | A24rv | CG_L_7_2 | 8 | -3,8,25 | Limbic |
| 55 | A6cdl | PrG_L_6_2 | 3 | -32,-9,58 | Frontal | **178** | A24rv | CG_R_7_2 | 8 | 5,22,12 | Limbic |
| 56 | A6cdl | PrG_R_6_2 | 3 | 33,-7,57 | Frontal | **179** | A32p | CG_L_7_3 | 7 | -6,34,21 | Limbic |
| 57 | A4ul | PrG_L_6_3 | 2 | -26,-25,63 | Frontal | **180** | A32p | CG_R_7_3 | 4 | 5,28,27 | Limbic |
| 58 | A4ul | PrG_R_6_3 | 2 | 34,-19,59 | Frontal | **181** | A23v | CG_L_7_4 | 7 | -8,-47,10 | Limbic |
| 59 | A4t | PrG_L_6_4 | 2 | -13,-20,73 | Frontal | **182** | A23v | CG_R_7_4 | 1 | 9,-44,11 | Limbic |
| 60 | A4t | PrG_R_6_4 | 2 | 15,-22,71 | Frontal | **183** | A24cd | CG_L_7_5 | 4 | -5,7,37 | Limbic |
| 61 | A4tl | PrG_L_6_5 | 4 | -52,0,8 | Frontal | **184** | A24cd | CG_R_7_5 | 4 | 4,6,38 | Limbic |
| 62 | A4tl | PrG_R_6_5 | 4 | 54,4,9 | Frontal | **185** | A23c | CG_L_7_6 | 4 | -7,-23,41 | Limbic |
| 63 | A6cvl | PrG_L_6_6 | 3 | -49,5,30 | Frontal | **186** | A23c | CG_R_7_6 | 4 | 6,-20,40 | Limbic |
| 64 | A6cvl | PrG_R_6_6 | 3 | 51,7,30 | Frontal | **187** | A32sg | CG_L_7_7 | 7 | -4,39,-2 | Limbic |
| 65 | A1/2/3ll | PCL_L_2_1 | 4 | -8,-38,58 | Frontal | **188** | A32sg | CG_R_7_7 | 7 | 5,41,6 | Limbic |
| 66 | A1/2/3ll | PCL_R_2_1 | 2 | 10,-34,54 | Frontal | **189** | cLinG | Cun_L_5_1 | 1 | -11,-82,-11 | Occipital |
| 67 | A4ll | PCL_L_2_2 | 2 | -4,-23,61 | Frontal | **190** | cLinG | Cun_R_5_1 | 1 | 10,-85,-9 | Occipital |
| 68 | A4ll | PCL_R_2_2 | 2 | 5,-21,61 | Frontal | **191** | rCunG | Cun_L_5_2 | 1 | -5,-81,10 | Occipital |
| 69 | A38m | STG_L_6_1 | 5 | -32,14,-34 | Temporal | **192** | rCunG | Cun_R_5_2 | 1 | 7,-76,11 | Occipital |
| 70 | A38m | STG_R_6_1 | 5 | 31,15,-34 | Temporal | **193** | cCunG | Cun_L_5_3 | 1 | -6,-94,1 | Occipital |
| 71 | A41/42 | STG_L_6_2 | 2 | -54,-32,12 | Temporal | **194** | cCunG | Cun_R_5_3 | 1 | 8,-90,12 | Occipital |
| 72 | A41/42 | STG_R_6_2 | 2 | 54,-24,11 | Temporal | **195** | rLinG | Cun_L_5_4 | 1 | -17,-60,-6 | Occipital |
| 73 | TE1.0/TE1.2 | STG_L_6_3 | 2 | -50,-11,1 | Temporal | **196** | rLinG | Cun_R_5_4 | 1 | 18,-60,-7 | Occipital |
| 74 | TE1.0/TE1.2 | STG_R_6_3 | 2 | 51,-4,-1 | Temporal | **197** | vmPOS | Cun_L_5_5 | 1 | -13,-68,12 | Occipital |
| 75 | A22c | STG_L_6_4 | 2 | -62,-33,7 | Temporal | **198** | vmPOS | Cun_R_5_5 | 1 | 15,-63,12 | Occipital |
| 76 | A22c | STG_R_6_4 | 2 | 66,-20,6 | Temporal | **199** | mOccG | OcG_L_4_1 | 1 | -31,-89,11 | Occipital |
| 77 | A38l | STG_L_6_5 | 5 | -45,11,-20 | Temporal | **200** | mOccG | OcG_R_4_1 | 1 | 34,-86,11 | Occipital |
| 78 | A38l | STG_R_6_5 | 5 | 47,12,-20 | Temporal | **201** | V5/MT | OcG_L_4_2 | 3 | -46,-74,3 | Occipital |
| 79 | A22r | STG_L_6_6 | 7 | -55,-3,-10 | Temporal | **202** | V5/MT | OcG_R_4_2 | 1 | 48,-70,-1 | Occipital |
| 80 | A22r | STG_R_6_6 | 7 | 56,-12,-5 | Temporal | **203** | OPC | OcG_L_4_3 | 1 | -18,-99,2 | Occipital |
| 81 | A21c | MTG_L_4_1 | 7 | -65,-30,-12 | Temporal | **204** | OPC | OcG_R_4_3 | 1 | 22,-97,4 | Occipital |
| 82 | A21c | MTG_R_4_1 | 6 | 65,-29,-13 | Temporal | **205** | iOccG | OcG_L_4_4 | 1 | -30,-88,-12 | Occipital |
| 83 | A21r | MTG_L_4_2 | 7 | -53,2,-30 | Temporal | **206** | iOccG | OcG_R_4_4 | 1 | 32,-85,-12 | Occipital |
| 84 | A21r | MTG_R_4_2 | 7 | 51,6,-32 | Temporal | **207** | msOccG | sOcG_L_2_1 | 1 | -11,-88,31 | Occipital |
| 85 | A37dl | MTG_L_4_3 | 3 | -59,-58,4 | Temporal | **208** | msOccG | sOcG_R_2_1 | 1 | 16,-85,34 | Occipital |
| 86 | A37dl | MTG_R_4_3 | 3 | 60,-53,3 | Temporal | **209** | lsOccG | sOcG_L_2_2 | 1 | -22,-77,36 | Occipital |
| 87 | aSTS | MTG_L_4_4 | 7 | -58,-20,-9 | Temporal | **210** | lsOccG | sOcG_R_2_2 | 1 | 29,-75,36 | Occipital |
| 88 | aSTS | MTG_R_4_4 | 7 | 58,-16,-10 | Temporal | **211** | mAmyg | Amyg_L_2_1 | 8 | -19,-2,-20 | Subcortical |
| 89 | A20iv | ITG_L_7_1 | 5 | -45,-26,-27 | Temporal | **212** | mAmyg | Amyg_R_2_1 | 8 | 19,-2,-19 | Subcortical |
| 90 | A20iv | ITG_R_7_1 | 5 | 46,-14,-33 | Temporal | **213** | lAmyg | Amyg_L_2_2 | 8 | -27,-4,-20 | Subcortical |
| 91 | A37elv | ITG_L_7_2 | 3 | -51,-57,-15 | Temporal | **214** | lAmyg | Amyg_R_2_2 | 8 | 28,-3,-20 | Subcortical |
| 92 | A37elv | ITG_R_7_2 | 3 | 53,-52,-18 | Temporal | **215** | rHipp | Hipp_L_2_1 | 8 | -22,-14,-19 | Subcortical |
| 93 | A20r | ITG_L_7_3 | 5 | -43,-2,-41 | Temporal | **216** | rHipp | Hipp_R_2_1 | 8 | 22,-12,-20 | Subcortical |
| 94 | A20r | ITG_R_7_3 | 5 | 40,0,-43 | Temporal | **217** | cHipp | Hipp_L_2_2 | 8 | -28,-30,-10 | Subcortical |
| 95 | A20il | ITG_L_7_4 | 7 | -56,-16,-28 | Temporal | **218** | cHipp | Hipp_R_2_2 | 8 | 29,-27,-10 | Subcortical |
| 96 | A20il | ITG_R_7_4 | 5 | 55,-11,-32 | Temporal | **219** | vCa | Str_L_6_1 | 8 | -12,14,0 | Subcortical |
| 97 | A37vl | ITG_L_7_5 | 3 | -55,-60,-6 | Temporal | **220** | vCa | Str_R_6_1 | 8 | 15,14,-2 | Subcortical |
| 98 | A37vl | ITG_R_7_5 | 3 | 54,-57,-8 | Temporal | **221** | GP | Str_L_6_2 | 8 | -22,-2,4 | Subcortical |
| 99 | A20cl | ITG_L_7_6 | 6 | -59,-42,-16 | Temporal | **222** | GP | Str_R_6_2 | 8 | 22,-2,3 | Subcortical |
| 100 | A20cl | ITG_R_7_6 | 6 | 61,-40,-17 | Temporal | **223** | NAC | Str_L_6_3 | 8 | -17,3,-9 | Subcortical |
| 101 | A20cv | ITG_L_7_7 | 5 | -55,-31,-27 | Temporal | **224** | NAC | Str_R_6_3 | 8 | 15,8,-9 | Subcortical |
| 102 | A20cv | ITG_R_7_7 | 5 | 54,-31,-26 | Temporal | **225** | vmPu | Str_L_6_4 | 8 | -23,7,-4 | Subcortical |
| 103 | A20rv | FuG_L_3_1 | 5 | -33,-16,-32 | Temporal | **226** | vmPu | Str_R_6_4 | 8 | 22,8,-1 | Subcortical |
| 104 | A20rv | FuG_R_3_1 | 5 | 33,-15,-34 | Temporal | **227** | dCa | Str_L_6_5 | 8 | -14,2,16 | Subcortical |
| 105 | A37mv | FuG_L_3_2 | 1 | -31,-64,-14 | Temporal | **228** | dCa | Str_R_6_5 | 8 | 14,5,14 | Subcortical |
| 106 | A37mv | FuG_R_3_2 | 1 | 31,-62,-14 | Temporal | **229** | dlPu | Str_L_6_6 | 8 | -28,-5,2 | Subcortical |
| 107 | A37lv | FuG_L_3_3 | 3 | -42,-51,-17 | Temporal | **230** | dlPu | Str_R_6_6 | 8 | 29,-3,1 | Subcortical |
| 108 | A37lv | FuG_R_3_3 | 1 | 43,-49,-19 | Temporal | **231** | mPFtha | Tha_L_8_1 | 8 | -7,-12,5 | Subcortical |
| 109 | A35/36r | PhG_L_6_1 | 5 | -27,-7,-34 | Temporal | **232** | mPFtha | Tha_R_8_1 | 8 | 7,-11,6 | Subcortical |
| 110 | A35/36r | PhG_R_6_1 | 5 | 28,-8,-33 | Temporal | **233** | mPMtha | Tha_L_8_2 | 8 | -18,-13,3 | Subcortical |
| 111 | A35/36c | PhG_L_6_2 | 5 | -25,-25,-26 | Temporal | **234** | mPMtha | Tha_R_8_2 | 8 | 12,-14,1 | Subcortical |
| 112 | A35/36c | PhG_R_6_2 | 1 | 26,-23,-27 | Temporal | **235** | Stha | Tha_L_8_3 | 8 | -18,-23,4 | Subcortical |
| 113 | TL | PhG_L_6_3 | 1 | -28,-32,-18 | Temporal | **236** | Stha | Tha_R_8_3 | 8 | 18,-22,3 | Subcortical |
| 114 | TL | PhG_R_6_3 | 1 | 30,-30,-18 | Temporal | **237** | rTtha | Tha_L_8_4 | 8 | -7,-14,7 | Subcortical |
| 115 | A28/34 | PhG_L_6_4 | 5 | -19,-12,-30 | Temporal | **238** | rTtha | Tha_R_8_4 | 8 | 3,-13,5 | Subcortical |
| 116 | A28/34 | PhG_R_6_4 | 5 | 19,-10,-30 | Temporal | **239** | PPtha | Tha_L_8_5 | 8 | -16,-24,6 | Subcortical |
| 117 | TI | PhG_L_6_5 | 5 | -23,2,-32 | Temporal | **240** | PPtha | Tha_R_8_5 | 8 | 15,-25,6 | Subcortical |
| 118 | TI | PhG_R_6_5 | 5 | 22,1,-36 | Temporal | **241** | Otha | Tha_L_8_6 | 8 | -15,-28,4 | Subcortical |
| 119 | TH | PhG_L_6_6 | 1 | -17,-39,-10 | Temporal | **242** | Otha | Tha_R_8_6 | 8 | 13,-27,8 | Subcortical |
| 120 | TH | PhG_R_6_6 | 1 | 19,-36,-11 | Temporal | **243** | cTtha | Tha_L_8_7 | 8 | -12,-22,13 | Subcortical |
| 121 | rpSTS | pSTS_L_2_1 | 7 | -54,-40,4 | Temporal | **244** | cTtha | Tha_R_8_7 | 8 | 10,-14,14 | Subcortical |
| 122 | rpSTS | pSTS_R_2_1 | 7 | 53,-37,3 | Temporal | **245** | lPFtha | Tha_L_8_8 | 8 | -11,-14,2 | Subcortical |
| 123 | cpSTS | pSTS_L_2_2 | 4 | -52,-50,11 | Temporal | **246** | lPFtha | Tha_R_8_8 | 8 | 13,-16,7 | Subcortical |

Note: 1: Visual network; 2: Somatomotor network; 3: Dorsal attention network; 4: Ventral attention network; 5: Limbic network; 6: Frontoparietal network; 7: Default mode network; 8: Subcortical network

Supplementary Table 3. The score performance of each task paradigm and the selected task performance.

| Task | Related task performance | Selected performance indicators |
| --- | --- | --- |
| **Social cognition** | Social_Task_Random_Perc_Random  Social_Task_Random_Median_RT_Random  Social_Task_TOM_Perc_TOM  Social_Task_TOM_Median_RT_TOM | Perc/ Median_RT |
| **Working Memory** | WM_Task_2bk_Acc  WM_Task_2bk_Median_RT | WM_Task_2bk_Acc /Median_RT |
| **Emotion processing** | Emotion_Task_Acc  Emotion_Task_Median_RT | Emotion_Task_Acc/Median_RT |
| **Gambling** | DDisc_AUC_40K  DDisc_AUC_200 | DDisc_AUC_40K - DDisc_AUC_200 |
| **Motor task** | Endurance Locomotion.  Dexterity Strength | Endurance |
| **Language** | Language_Task_Story_Acc  Language_Task_Math_Acc | Language_Task_Story_Acc |
| **Relational processing** | PicVocab_Unadj | PicVocab_Unadj |
| **Rest fMRI** | PicVocab ReadEng PicSeqFlanker  CardSort ProcSpeed  PMAT24_A_CR VSPLOT_TC IWRD_TOT  ListSort SCPT_Eff DDisc | Confirmatory Factor Analysis |

Supplementary Table 4. The influence of different classification models on the classification results of ADHD when the optimal task combination is adopted. The classification effects of four, five, and six combinations.

| Task Group  Method | M_R_S_W | | M_R_S_G_E | | G_L_M_R_S_W | |
| --- | --- | --- | --- | --- | --- | --- |
|  | AUC | ACC | AUC | ACC | AUC | ACC |
| KNN | 0.562 | 0.562 | 0.567 | 0.569 | 0.544 | 0.562 |
| Extra Tree | 0.533 | 0.532 | 0.563 | 0.564 | 0.460 | 0.469 |
| Adaboost | 0.578 | 0.579 | 0.559 | 0.560 | 0.526 | 0.550 |
| Gauss DB | 0.587 | 0.584 | 0.578 | 0.573 | 0.533 | 0.539 |
| Gradient Boost | 0.577 | 0.577 | 0.529 | 0.532 | 0.544 | 0.562 |
| SVM | 0.558 | 0.558 | 0.536 | 0.536 | 0.561 | 0.568 |
| Voting | 0.566 | 0.566 | 0.549 | 0.549 | 0.548 | 0.554 |
| DT | 0.508 | 0.508 | 0.532 | 0.532 | 0.534 | 0.540 |
| MLP | 0.497 | 0.486 | 0.500 | 0.502 | 0.507 | 0.462 |
| CNN | 0.532 | 0.539 | 0.501 | 0.517 | 0.554 | 0.566 |
| MAGE | 0.491 | 0.512 | 0.456 | 0.510 | 0.424 | 0.460 |
| EVGCN | 0.575 | 0.615 | 0.584 | 0.623 | 0.588 | 0.594 |
| TSP-GNN | 0.724 | 0.671 | 0.721 | 0.656 | 0.720 | 0.680 |

Supplementary Table 5. The influence of different classification models on the classification results of ABIDE when the optimal task combination is adopted. The classification effects of four, five, and six combinations.

| Task Group  Method | G_E_S_W | | G_E_S_M_L | | G_E_S_W_L_M | |
| --- | --- | --- | --- | --- | --- | --- |
|  | AUC | ACC | AUC | ACC | AUC | ACC |
| KNN | 0.553 | 0.576 | 0.560 | 0.584 | 0.544 | 0.562 |
| Extra Tree | 0.526 | 0.542 | 0.531 | 0.548 | 0.460 | 0.469 |
| Adaboost | 0.494 | 0.520 | 0.527 | 0.556 | 0.526 | 0.554 |
| Gauss DB | 0.609 | 0.618 | 0.592 | 0.598 | 0.533 | 0.539 |
| Gradient Boost | 0.519 | 0.542 | 0.561 | 0.582 | 0.544 | 0.562 |
| SVM | 0.567 | 0.573 | 0.588 | 0.593 | 0.561 | 0.568 |
| Voting | 0.558 | 0.567 | 0.562 | 0.570 | 0.548 | 0.554 |
| DT | 0.499 | 0.506 | 0.519 | 0.522 | 0.534 | 0.540 |
| MLP | 0.539 | 0.542 | 0.574 | 0.574 | 0.589 | 0.588 |
| CNN | 0.560 | 0.566 | 0.515 | 0.537 | 0.518 | 0.520 |
| MAGE | 0.445 | 0.458 | 0.524 | 0.447 | 0.496 | 0.472 |
| EVGCN | 0.598 | 0.624 | 0.598 | 0.618 | 0.584 | 0.596 |
| TSP-GNN | 0.759 | 0.702 | 0.741 | 0.705 | 0.728 | 0.694 |

# Supplementary Figures


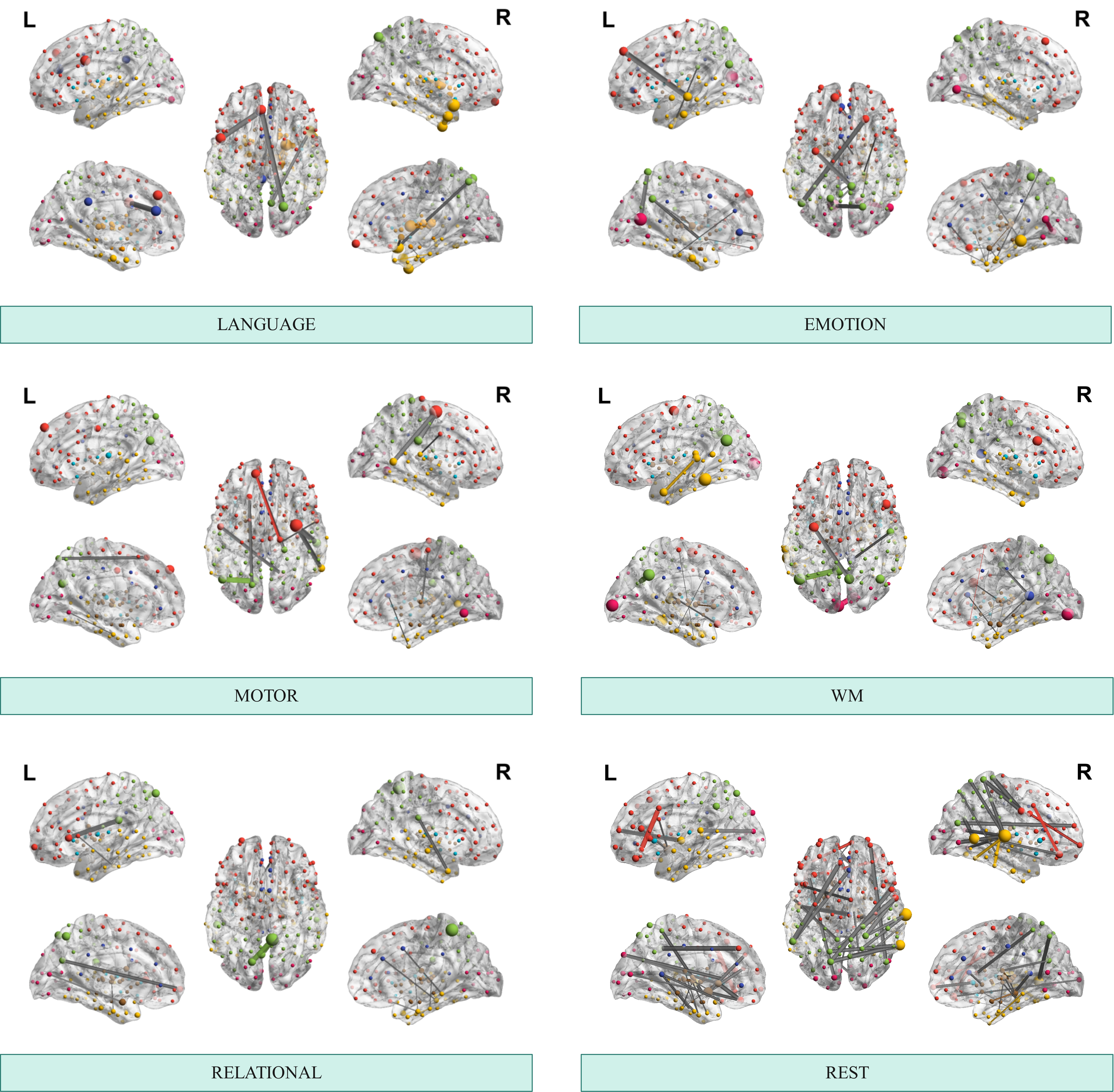


Supplementary Figure 1. The distribution of edges was decoded from five task states and a resting-state fMRI and plotted through BrainNetViewer.


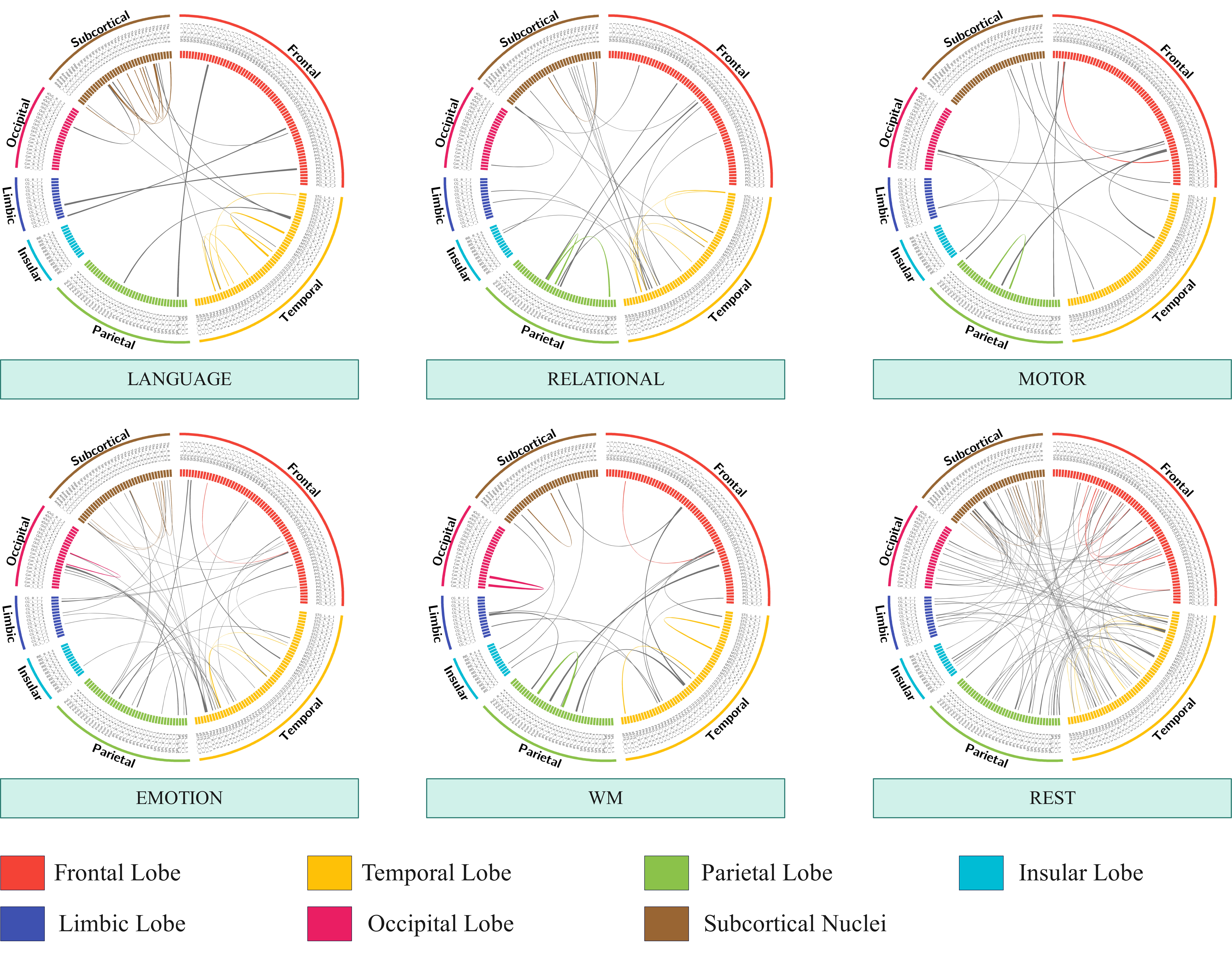


Supplementary Figure 2. The distribution of edges was decoded from five task states and a resting state fMRI and plotted through circos plot.

***References***

Binder, J.R., Gross, W.L., Allendorfer, J.B., Bonilha, L., Chapin, J., Edwards, J.C., et al. (2011). Mapping anterior temporal lobe language areas with fMRI: a multicenter normative study. *Neuroimage* 54(2)**,** 1465-1475. doi: 10.1016/j.neuroimage.2010.09.048.

Buckner, R.L., Krienen, F.M., Castellanos, A., Diaz, J.C., and Yeo, B.T. (2011). The organization of the human cerebellum estimated by intrinsic functional connectivity. *J Neurophysiol* 106(5)**,** 2322-2345. doi: 10.1152/jn.00339.2011.

Castelli, F., Frith, C., Happé, F., and Frith, U. (2002). Autism, Asperger syndrome and brain mechanisms for the attribution of mental states to animated shapes. *Brain* 125(8)**,** 1839-1849. doi: 10.1093/brain/awf189.

Delgado, M.R., Nystrom, L.E., Fissell, C., Noll, D.C., and Fiez, J.A. (2000). Tracking the hemodynamic responses to reward and punishment in the striatum. *J Neurophysiol* 84(6)**,** 3072-3077. doi: 10.1152/jn.2000.84.6.3072.

Gevins, A., and Cutillo, B. (1993). Spatiotemporal dynamics of component processes in human working memory. *Electroencephalography and Clinical Neurophysiology* 87(3)**,** 128-143. doi: <https://doi.org/10.1016/0013-4694(93)90119-G>.

Manuck, S.B., Brown, S.M., Forbes, E.E., and Hariri, A.R. (2007). Temporal stability of individual differences in amygdala reactivity. *American Journal of Psychiatry* 164(10)**,** 1613-1614.

Smith, R., Keramatian, K., and Christoff, K. (2007). Localizing the rostrolateral prefrontal cortex at the individual level. *Neuroimage* 36(4)**,** 1387-1396.

White, S.J., Coniston, D., Rogers, R., and Frith, U. (2011). Developing the Frith-Happé animations: A quick and objective test of Theory of Mind for adults with autism. *Autism Research* 4(2)**,** 149-154. doi: <https://doi.org/10.1002/aur.174>.
